# Wnt10b-overexpressing umbilical cord mesenchymal stem cells promote fracture healing via accelerated cartilage callus to bone remodeling

**DOI:** 10.1080/21655979.2022.2062954

**Published:** 2022-04-18

**Authors:** Yuxiang Hu, Yu He, Jiarui Fang, Yunlu Liu, Yulin Cao, Wei Tong, Wei Chen, Zengwu Shao, Yong Liu, Hongtao Tian

**Affiliations:** a Department of Orthopedics, Union Hospital, Tongji Medical College, Huazhong University of Science and Technology, Wuhan Hubei, China; b Department of Orthopedics, The Third Hospital of Hebei Medical University, Shi Jiazhuang, Hebei, China; c Nhc Key Laboratory of Intelligent Orthopedic Equipment (The Third Hospital of Hebei Medical University), Shi Jiazhuang, Hebei, China

**Keywords:** Bone regeneration, umbilical cord MSC, osteogenesis and angiogenesis, wnt signaling pathway

## Abstract

The aim of this study was to investigate whether HUCMSCs^Wnt10b^ could promote long bone fracture healing. Commercially-available HUCMSCs^Emp^ (human umbilical cord mesenchymal stem cells transfected with empty vector) in hydrogel, HUCMSCs^Wnt10b^ in hydrogel and HUCMSCs^Wnt10b^ with the Wnt signaling pathway inhibitor IWR-1 were transplanted into the fracture site in a rat model of femoral fracture. We found that transplantation of HUCMSCs^Wnt10b^ significantly accelerated bone healing in a rat model of femoral fracture. Meanwhile, three-point bending test proved that the mechanical properties of the bone at the fracture site in the HUCMSC^Wnt10b^ treatment group were significantly better than those of the other treatment groups. To understand the cellular mechanism, we explored the viability of periosteal stem cells (PSCs), as they contribute the greatest number of osteoblast lineage cells to the callus. In line with *in vivo* data, we found that conditioned medium from HUCMSCs^Wnt10b^ enhanced the migration and osteogenic differentiation of PSCs. Furthermore, conditioned medium from HUCMSCs^Wnt10b^ also induced endothelial cells to form capillary-like structures in a tube formation assay, which was blocked by *SU5416*, an angiogenesis inhibitor, suggesting that enhanced vessel formation and growth also contribute to accelerated hard callus formation. In summary, our study demonstrates that HUCMSCs^Wnt10b^ promote fracture healing via accelerated hard callus formation, possibly due to enhanced osteogenic differentiation of PSCs and vessel growth. Therefore, HUCMSCs^Wnt10b^ may be a promising treatment for long bone fractures.

## Grapical abstract

**Figure:**
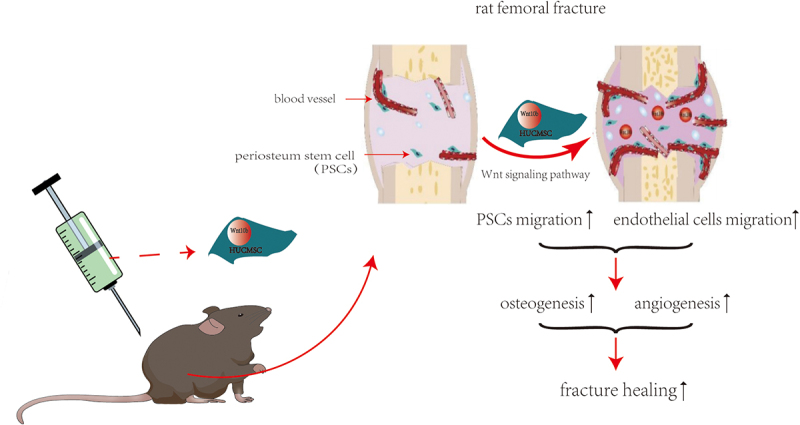

## Research highlights

• We constructed novel transgenic HUCMSCs for bone regeneration

• HUCMSCs^Wnt10b^ significantly promoted healing in a rat femoral fracture model

• HUCMSCs^Wnt10b^ accelerated turnover of cartilage callus to hard callus

• HUCMSCs^Wnt10b^ showed strong osteogenic and angiogenic effects.

## 1 Introduction

Approximately five million patients experience a fracture each year in China, of whom 3–7% suffer delayed healing or nonunion fractures [1]. These failures are a substantial physical, medical and financial burden for both individuals and healthcare systems [2]. Consequently, further clarification of the pathological mechanism of delayed union or nonunion of fractures in the fracture microenvironment under pathological conditions is a significant issue for both surgeons and basic scientists.

Long bone fracture healing is a complex physiological process known as endochondral ossification, which is divided into three stages: the inflammatory hematoma formation period, the callus formation period and the bone remodeling period [3]. This sequence of events is totally different from that of flat bones such as the calvaria, were new bone is formed via intramembranous ossification. At each stage, this continuous change in cellular function is precisely regulated by a series of signal transduction pathways, such as Wingless/Integrated (Wnt)/β-catenin, bone morphogenetic protein (BMP)/transforming growth factor beta (TGF-β), etc [4]. During fracture healing, the callus provides a microenvironment that promotes bone formation and maintains temporary biomechanical stability [5]. Consequently, callus formation disorder is an important cause of atrophic fracture nonunion [6,7]. The osteogenic lineage cells in the callus are mainly derived from periosteal stem cells (PSCs), so impairment of PSC function is an important cause of bone regeneration disorders [8–10]. The Wnt signaling pathway has important biological functions during bone regeneration [11,12], and changes in serum Wnt protein levels are highly correlated with fracture healing progress [13]. We previously found that Wnt10b-overexpressing human umbilical cord mesenchymal stem cells (HUCMSCs^Wnt^ [10]b) significantly promoted calvarial defect healing through enhanced osteogenesis and angiogenesis. However, due to the totally different mechanism of long bone fracture healing, whether Wnt10b-overexpressing HUCMSCs are a suitable treatment for fracture healing in long bones is still unknown.

We hypothesized that HUCMSCs^Wnt^ [10]b could promote fracture healing by enhancing the function of both PSCs and vascular endothelial cells. Therefore, in this study, we generated HUCMSCs^Wnt^ [10]b to explore their effects on femoral fracture healing in a rat model. Our study may provide a promising cell source for fracture healing.

## 2 Materials and methods

### 2.1 Construction of HUCMSCs^Wnt^ *[10]*b

HUCMSCs (human umbilical cord mesenchymal stem cells) were purchased from Wuhan Hamilton Biotechnology Co., LTD, and inoculated into 6-well plates at 4 × 10^6^ cells/well. When cells reached 90% confluence in each well, the culture medium (Huxuc-03061; Cyagen Biosciences Inc., Guangzhou, China) was changed and lentiviral vector was used to introduce the Wnt10b gene to construct transgenic umbilical cord mesenchymal stem cells (HUCMSCs^Wnt^ [10]b); umbilical cord mesenchymal stem cells transfected with empty vector (HUCMSCs^Emp^) were used as controls. Both were provided by the technical department of Wuhan Hamilton Biotechnology Co., Ltd [14].

### 2.2 Rat femoral fracture model

All animal experiments in this study followed the guiding principles of the Animal Welfare Law and were approved by the Animal Care and Use Committee of the Union Hospital of Huazhong University of Science and Technology (Ethical approval number: 2644). Fractures were surgically created in 8 weeks-old Sprague-Dawley (SD) rats at different time points (1, 2, 4 and 6 weeks, n = 8 in each group at each time point), and the following treatments are introduced separately: (A) saline (veh) (B) hydrogel (gel) (C) hydrogel with HUCMSCs^Emp^ (D) hydrogel with HUCMSC^Wnt^ [10]b (E) hydrogel with HUCMSC^Wnt^ [10]b + IWR-1. Rats were anesthetized by injection with Amobarbital Sodium (10%, 0.5 mL/100 g). A Kirschner wire was inserted into the upper end of the femur for internal fixation, then the midshaft of the femur was cut to create a femoral fracture model. The wound was disinfected and sutured, and antibiotics were given for the first three days after operation to combat infection. Hydrogel was used to support the cells locally and prevent cells leakage; cells (1 × 10^5^) were mixed with an equivalent volume of commercially-available hydrogel (BD™, Puramatrix™ catalog number 354,250; BD Biosciences, Franklin Lakes, NJ, USA) and placed at the fracture site [14,15].

### 2.3 Micro-CT analysis

Femurs were collected at 1, 2, 4 and 6 weeks after surgery, and fixed with 4% paraformaldehyde for 48 hours. Then a SkyScan 1176 high-resolution micro-CT imaging system (micro-CT) was used to scan the femurs at 18 μm resolution, with a 1 mm aluminum filter, 90 kV voltage and 273 μA current. The raw data obtained were processed using SkyScan NRecon reconstruction software and CT-VOX three-dimensional reconstruction analysis software for volume reconstruction and three-dimensional image generation. Bone volume fraction (BT/TV) was determined by three-dimensional standard microstructure analysis. Based on micro-CT images, fracture healing scores were calculated from 4-week fracture samples based on an improved five-point X-ray scoring system [10].

### 2.4 Three-point bending test

The stored bone specimens were equilibrated at room temperature then placed across two fulcrums 5 mm apart. An axial load was then applied to the fractured part between the two fulcrums. The load was applied at a rate of 5 mm/min until the bone broke. This test was performed with a universal three-point bending machine. The load-failure curve was recorded and the max-load, max-stress and elastic modulus were quantified [16].

### 2.5 Histology and immunohistochemistry

The rat femurs were collected and fixed with 4% paraformaldehyde for 24 hours, and then decalcified at room temperature with 10% EDTA for 30 days. After that, the specimens were dehydrated with a graded series of ethanol concentrations and embedded in paraffin. After embedding, the paraffin-embedded specimens were cut into sagittal sections at a thickness of 6 μm, and stained with safranin O-fast green.

Paraffin sections were also used for immunohistochemistry after appropriate antigenic repair and slides were incubated overnight at 4°C with antibodies against CD31(1:100, BD, 555025), and osteocalcin (OCN; 1:100, SC-365797). Next day, the secondary antibody was applied, and the results was visualized with DAB color solution. After color development was complete, the slides were counterstained with hematoxylin for 2 minutes, then dehydrated with 100% anhydrous ethanol, coverslipped and sealed. This experiment was conducted by Hubei Baos Biotechnology Co., Ltd., which provided all the reagents except the primary antibodies.

### 2.6 Conditioned medium collection

HUCMSCs^Emp^ and HUCMSC^Wnt^ [10]b were seeded into 6-well plates and cultured until they reached 80%–90% confluence, then washed three times with phosphate-buffered saline and changed to serum-free α-MEM. Next day the conditioned medium was collected and centrifuged at 2,000 rpm for 5 minutes to remove cell debris before the medium was used in experiments [17].

### 2.7 Cell migration experiment

Periosteal stem cells were plated into the upper chamber of a 24-well Transwell™ plate at a density of 20,000 cells/well. HUCMSCs^Emp^ -conditioned medium and HUCMSC^Wnt^ [10]b-conditioned medium were added to the lower chamber. After culture for 24 hours, the cells on the bottom surface of each Transwell™ were fixed with 4% paraformaldehyde and stained with crystal violet, then the number of cells that had migrated was observed under a microscope.

### 2.8 Alkaline phosphatase (ALP) and alizarin red (ARS) staining

Periosteal stem cells were seeded into 6-well plates, and when the cell density reached 80–90%, they were changed to culture in osteogenic differentiation medium containing different treatments (control group and HUCMSC^Wnt^ [10]b group). After 14 days, the cells were fixed and an ALP staining kit was used to detect ALP activity. ARS staining was performed after 28 days of treatment to detect mineralization, and the staining was observed and analyzed using Image J (NIH, Bethesda, MD, USA).

### 2.9 Real-time polymerase chain reaction (RT-PCR)

Total RNA was extracted by the TRIzol method, and cDNA was synthesized by reverse transcription using a high-capacity cDNA reverse transcription kit (Applied Biosystems, Foster City, CA, USA). A PCR machine was used for RT-PCR detection. Expression levels were normalized to GAPDH which was used as the internal reference. The 2^−ΔΔCT^ method was used to analyze the relative expression levels of each target gene. The primer sequences used in this study were as follows: *ALP* (Forward: 5ʹ-GGGGACATGCAGTATGAATT-3ʹ, Reverse: 5ʹ- GGCCTGGTAGTTGTTGTGAG-3ʹ); *RUNX2* (Forward: 5ʹ- CAGACCAGCAGCACTCCATA-3ʹ, Reverse: 5ʹ- AGACTCATCCATTCTGCCGC-3ʹ); *Osx* (Forward: 5ʹ- CCCACCCTTCCCTCACTCAT-3ʹ, Reverse: 5ʹ-CCTTGTACCACGAGCCATAGG-3ʹ), *GAPDH* (Forward: 5ʹ- ACCACGAGAAATATGACAACTCCC-3ʹ, Reverse: 5ʹ- CCAAAGTTGTCATGGATGACC-3ʹ).

### 2.10 Western blot assay

For total protein extraction, cells were lysed in RIPA buffer (Thermo Fisher Scientific, Waltham, MA, USA) supplemented with 1% Protease inhibitor on ice. Protein concentration was determined by the BCA assay (Sigma-Aldrich, St Louis, MO, USA). Next, proteins were subjected to sodium dodecyl sulfate polyacrylamide gel electrophoresis (SDS-PAGE) and transferred to a 0.2 μm polyvinylidene difluoride membrane. The membranes were blocked with 5% skimmed milk and incubated with the primary antibodies anti-*RUNX2* (Abcam, Cambridge, MA, USA ab236639) and anti-*OSTERIX* (Abcam, ab209484) overnight at 4°C. Glyceraldehyde 3-phosphate dehydrogenase (*GAPDH*, Abcam, ab8245) was used as an internal reference. Next day, secondary antibodies against the species of primary antibodies were added and incubated for 1 hour at room temperature. Signals on the membrane were detected by electrochemiluminescence (ECL, Pierce, Rockford, IL, USA), and the protein band density of immune complexes was analyzed using AlphaEaseFC (Alpha Innotech Corporation, San Leandro, CA, USA) software.

### 2.11 Angiogenesis experiment

Vascular matrix gel was added to a 96-well plate on ice at a volume of 50 μL per well, then the 96-well plate was placed at 37°C for 1 hour to allow gelation. Then human umbilical vein endothelial cells (HUVECs) were added at a density of 20,000 cells/well, and the plate was placed in a 37°C, 5% CO2 tissue culture incubator. After 4 hours, angiogenesis was observed under a light microscope, and the number of lumens and branch points formed was counted.

### 2.12 Statistical analysis

Descriptive statistical results are expressed in the form of mean ± SEM. All statistical analysis and mapping were performed with GraphPad Prism 8.0. Groups were compared using t-test (paired or unpaired as appropriate), or one-way or two-way analysis of variance (repeated measurements as appropriate) and Bonferroni correction was used for analysis to adjust for multiplicity. *P* values of less than 0.05 were considered statistically significant.

## 3 RESULTS

The objective of this study was to evaluate the effect of HUCMSCs^Wnt^ [10]b on fracture healing and investigate the underlying mechanism, we first established a rat model of femoral fracture then treated the fracture with saline, hydrogel, hydrogel with HUCMSCs^Emp^, hydrogel with HUCMSCs^Wnt^ [10]b or hydrogel with HUCMSCs^Wnt^ [10]b + IWR-1. Subsequently, micro-CT analysis, three-point bending tests and histological analysis were performed to evaluate the therapeutic effect of HUCMSCs^Wnt^ [10]b. Lastly, we verified the effect of HUCMSCs^Wnt^ [10]b conditioned medium on the PSCs and HUVECs *in vitro* and concluded that HUCMSCs^Wnt^ [10]b significantly promoted fracture healing by accelerating the turnover of cartilage to bone.

### 3.1 HUCMSCs^Wnt^ *[10]*b accelerated fracture healing in a femoral fracture rat model

**Figure 1.**
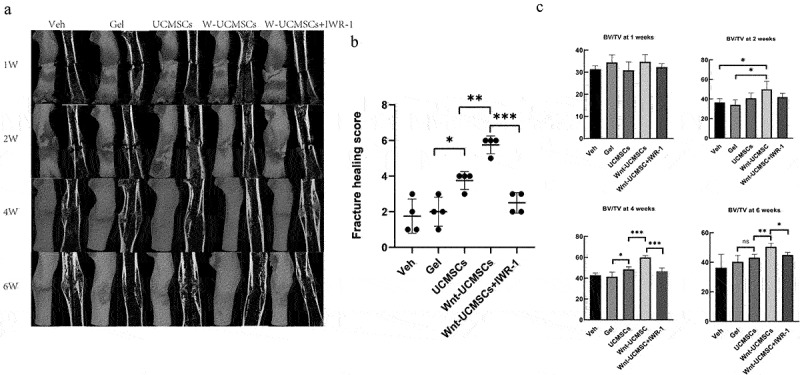
HUCMSCs^Wnt10b^ promoted the healing of a femoral fracture in a rat model. (a) Rat femoral fracture was treated with veh, hydrogel, hydrogel with HUCMSCs^Emp^, hydrogel with HUCMSCs^Wnt10b^ or hydrogel with HUCMSCs^Wnt10b^ + IWR-1 (the Wnt signaling pathway inhibitor), then femurs were harvested at 1, 2, 4 and 6 weeks after surgery. Three-dimensional reconstruction showed the healing of the fracture. (b) Fracture healing scores were quantified based on micro-CT images at 4 weeks after surgery. (c) Micro-CT analysis of the BV/TV of the fracture area at 1, 2, 4 and 6 weeks after surgery (n = 8, mean ± SEM, *: *P* < 0.05, **: *P* < 0.01, ***: *P* < 0.001, Student’s t test was used for statistical analysis).

We incorporated HUCMSCs^Emp^ and HUCMSCs^Wnt^ [10]b into a commercially-available hydrogel to provide a physically steady environment for the cells. Next, we injected the cell–hydrogel complex into the fracture site, and then micro-CT scanning was performed at 1, 2, 4 and 6 weeks post-surgery. The results showed that the calluses at the fracture site were still obvious after 4 weeks in the veh- and gel-treated groups. However, in the HUCMSCs^Emp^ treatment group, the callus was smaller than in the former two groups, but the fracture site was still clearly visible. Notably, in the HUCMSCs^Wnt^ [10]b treatment group, the callus had almost disappeared, leaving an almost nearly normal femur and a significantly better fracture healing score (Figure 1b). The effects were significantly reduced by the addition of IWR-1, a Wnt signaling pathway inhibitor (Figure 1(a,b)). Regarding the bone volume fraction, the veh- and gel-treated groups had similar bone volume fraction at the 4 w time-point: 42 ± 0.26% and 40 ± 0.33%, respectively; both lower than the HUCMSCs^Emp^ treatment group (49 ± 0.28%). However, the bone volume in the HUCMSCs^Wnt^ [10]b treatment group was 17 ± 0.24% and 10 ± 0.17% higher than in the veh and HUCMSCs^Emp^ groups, respectively, and these effects were abolished by the treatment with IWR-1 (Figure 1c). These results suggested that HUCMSCs^Wnt^ [10]b promoted fracture healing via enhanced Wnt signaling.

### 3.2 HUCMSCs^Wnt^ *[10]*b improved the mechanical properties of the repaired femur

**Figure 2.**
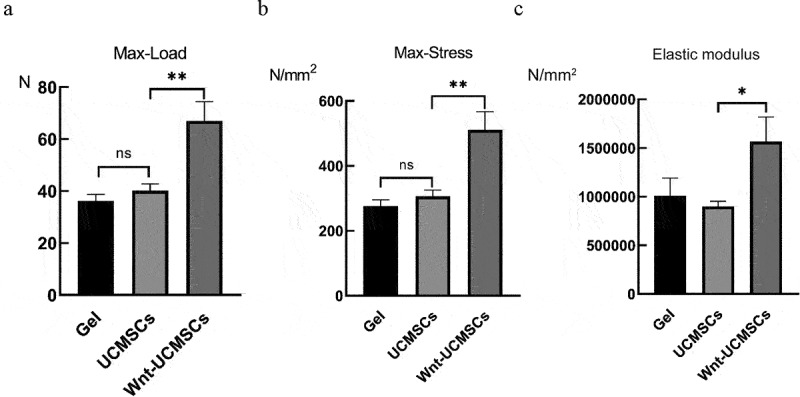
The UCMSC^Wnt10b^ treatment group exhibited better mechanical properties of the healing femoral fracture. (A, B and C) The maximum load, maximum stress and elastic modulus at failure of the repaired fracture were calculated from the load and displacement curves (n = 8, mean ± SEM, *: *P* < 0.05, **: *P* < 0.01, Student’s t test was used for statistical analysis).

As mechanical properties are a key indicator of long bone fracture healing, the femurs were collected at 4 weeks after surgery and subjected to a three-point bending test. As can be seen from the results, max-load, max-stress and elastic modulus in the femurs did not differ significantly between the hydrogel-treated groups and the HUCMSCs^Emp^-treated group, while in the HUCMSCs^Wnt^ [10]b-treated group, the max-load, max-stress and elastic modulus increased by 70%, 93% and 50% respectively, compared with the two groups, which proved that the mechanical properties of the healed femoral fracture in the HUCMSCs^Wnt^ [10]b treatment group were significantly better than those in the other groups (Figure 2a, b and c).

### 3.3 HUCMSCs^Wnt^ *[10]*b induced accelerated turnover of cartilage callus to hard callus

**Figure 3.**
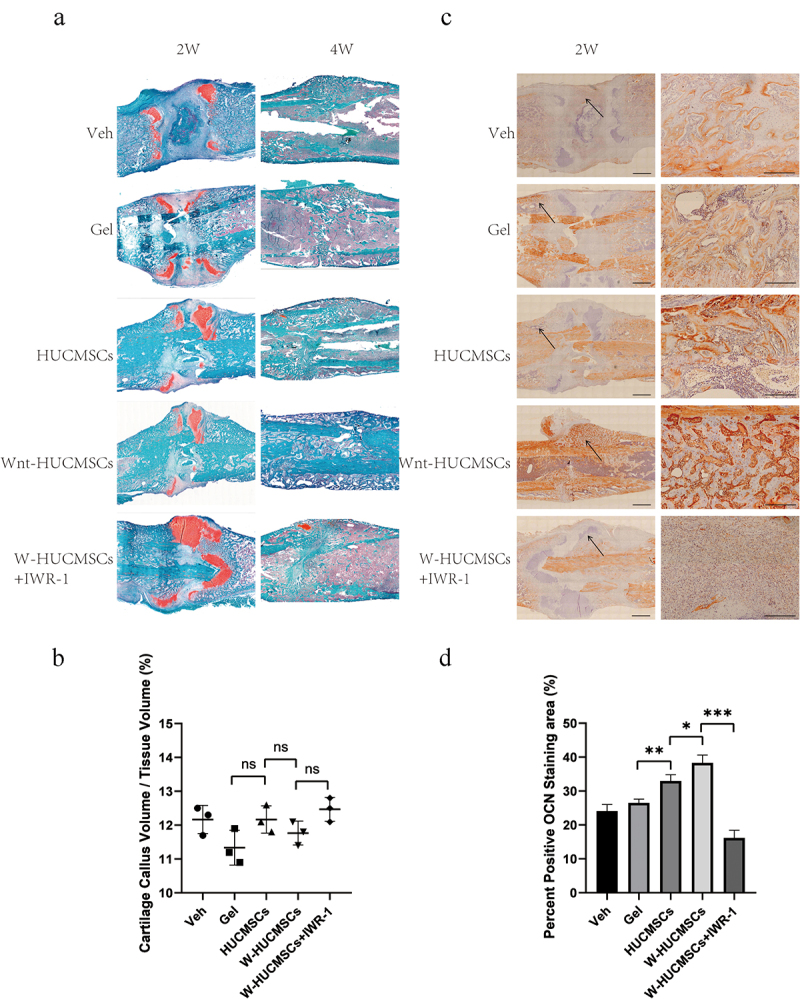
Histological analysis of femurs during fracture healing. (a) Sections were stained with safranin O–fast green and labeled for cartilage (red) and mineralized tissue (green) at 2 and 4 weeks after surgery. (b) Based on histology, cartilage callus volume/tissue volume was assessed using Image J at 2 weeks after surgery. (c) Immunohistochemistry was used to identify the expression of OCN in the callus of the fracture site at 2 weeks after surgery. On the right is an enlarged image of the area indicated by the arrow, which is the interface between the bone and the callus. (d) The area of positive OCN staining was analyzed using Image J. (n = 3, mean ± SEM, ns: not significant, *: *P* < 0.05, **: *P* < 0.01, ***: *P* < 0.001, Student’s t test, results are representative of at least three independent experiments, scale bar = 1 mm (left picture), scale bar = 200 μm (zoom in image on the right picture)).

In order to clarify the possible mechanism of action of HUCMSCs^Wnt^ [10]b on fracture healing, we examined the callus by histology. Interestingly, we found similar percentages of cartilage callus at 2 weeks in the HUCMSCs^Wnt^ [10]b-treated rats and the controls (Figure 3b), but significantly more bone formation in HUCMSCs^Wnt^ [10]b treated callus at 4 weeks, indicating an accelerated turnover of cartilage callus to hard callus (Figure 3a). This could be due to enhancement of the osteogenic differentiation, as much stronger OCN staining was also seen in the HUCMSCs^Wnt^ [10]b treatment group (Figure 3(c,d)). Furthermore, these effects were largely abolished by IWR-1 (Figure 3a, c and d). In summary, the effects of HUCMSCs^Wnt^ [10]b on fracture healing may be due to accelerated cartilage remodeling and hard callus formation.

### 3.4 HUCMSCs^Wnt^ *[10]*b promoted the migration and osteogenic differentiation of PSCs in vitro

**Figure 4.**
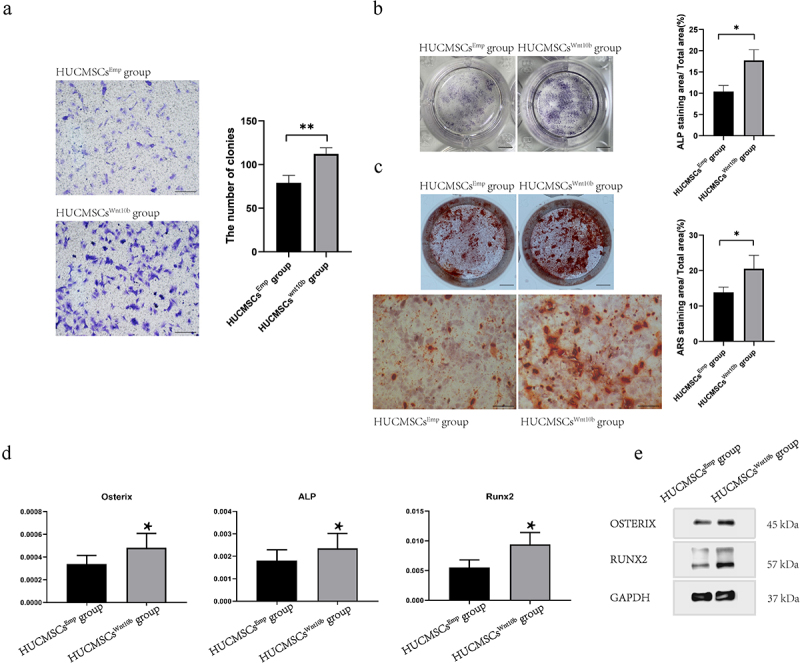
HUCMSCs^Wnt10b^ enhanced the migration and osteogenic differentiation of periosteal stem cells (PSCs). (a) The migration ability of PSCs was assessed by Transwell™ assay. PSCs were seeded into the upper chambers of Transwell™ at 2,000 cells/mL, then HUCMSCs^Emp^-conditioned medium or HUCMSCs^Wnt10b^-conditioned medium was added to the lower chamber. After 24 hours, cells on the lower surface of the Transwell™ membrane were stained with crystal violet. The number of colonies was quantified using Image J (n = 3, mean ± SEM, **: *P* < 0.01, by Student’s t test, results are representative of at least three independent experiments, scale bar = 200 μm). (b) PSCs were treated with HUCMSCs^Emp^-conditioned medium or HUCMSCs^Wnt10b^-conditioned medium and stained for alkaline phosphatase (ALP) at 7 days. The area of ALP staining was analyzed using ImageJ. (scale bar = 0.5 μm). (c) PSCs were treated with HUCMSCs^Emp^-conditioned medium or HUCMSCs^Wnt10b^-conditioned medium, then used for alizarin red (ARS) staining at 28 days. Plate view (top) and microscopic view (bottom) of ARS staining are shown. In addition, the area of mineralization was measured using ImageJ. (scale bar = 0.5 μm (top picture), scale bar = 200 μm (bottom picture)). (d) Expression of the osteogenic genes *ALP, RUNX2* and *Osterix* was assessed by RT-PCR. (e) The expression of osteogenic-related proteins (*RUNX2* and *Osterix*) in PSCs was detected by western blotting. (n = 3, mean ± SEM, *: *P* < 0.05, **: *P* < 0.01, by Student’s t test, results are representative of at least three independent experiments).

As PSCs constitute most of the osteoblast lineage cells in the fracture callus, we next explored the modulating effects of HUCMSCs^Wnt^ [10]b on PSCs so as to understand the cellular mechanism. We performed Transwell™ assays, and found that compared with the HUCMSCs^Emp^-treated group, the HUCMSCs^Wnt^ [10]b-treated group significantly promoted the migration of PSCs when conditioned medium from HUCMSCs^Wnt^ [10]b was added to the lower chambers of a Transwell™ plate (Figure 4a). Next, ALP staining and APS staining were performed to study whether HUCMSCs^Wnt^ [10]b conditioned medium modulated the osteogenic differentiation ability of PSCs. As can be seen from the results, HUCMSCs^Wnt^ [10]b conditioned medium significantly enhanced both ALP activity (Figure 4b) and ARS staining (Figure 4c). Moreover, RT-PCR revealed a significant increase in *ALP, Runx2* and *Osterix* gene expression in HUCMSCs^Wnt^ [10]b-treated PSCs (Figure 4d). To further confirm this change at the protein level, total protein was collected and western blot analysis was performed. The results showed that, compared with the control group, the expression of *Runx2* and *Osterix* protein in HUCMSCs^Wnt^ [10]b-treated PSCs were significantly increased (Figure 4e). In summary, HUCMSCs^Wnt^ [10]b enhanced both migration and osteogenic differentiation ability of PSCs, which is a possible mechanism underlying the accelerated hard callus formation and thus promoting fracture healing.

### 3.5 HUCMSCs^Wnt^ *[10]*b promoted angiogenesis and migration of HUVECs

**Figure 5.**
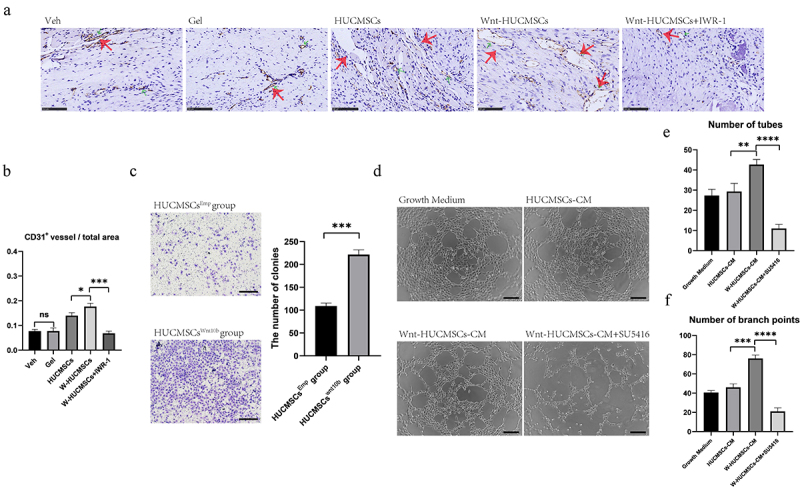
HUCMSCs^Wnt10b^ promoted the migration and angiogenesis of HUVECs. (a) Immunohistochemistry was used to identify the expression of CD31 in the callus of the fracture site at 2 weeks after surgery. (b) The number of CD31^+^ vessels in the total area was analyzed using Image J (n = 8, the red arrows indicate the CD31^+^ blood vessels, scale bar = 200 μm). (c) Transwell™ assay was performed to analyze the migration ability of the HUVECs. HUVECs were seeded into the upper chambers at 2,000 cells/mL, and HUCMSCs^Emp^-conditioned medium or HUCMSCs^Wnt10b^-conditioned medium was added to the lower chambers. After 24 hours, cells on the lower surface of the Transwell™membrane were stained with crystal violet. The number of colonies was quantified using Image J (n = 3, mean ± SEM, ***: *P* < 0.001, by Student’s t test, results are representative of at least three independent experiments, scale bar = 200 μm). (d) HUVECs were seeded into a 96-well plate and treated with growth medium, HUCMSCs-CM, Wnt-HUCMSCs-CM or Wnt-HUCMSCs+*SU5416* (an angiogenesis inhibitor). Four hours later, pictures were taken under a microscope. (e, f) The number of tubes (e) and branch points (f) were counted (n = 3, mean ± SEM, **: *P* < 0.01, ****: *P* < 0.0001, by Student’s t test, results are representative of at least three independent experiments, scale bar = 100 μm).

Vessel growth plays a vital role in the turnover of cartilage to bone both in the growth plate during development and in fracture healing. We previously found that HUCMSCs^Wnt^ [10]b enhanced angiogenesis, so to evaluate the involvement of this activity in fracture healing we performed CD31 immunostaining for blood vessels at 2 weeks after surgery. The results showed that CD31-positive areas were significantly increased in the callus of HUCMSCs^Wnt^ [10]b-treated rats compared with the other groups (Figure 5a). In addition, the number of CD31^+^ vessels in the total area was obviously higher in the HUCMSCs^Wnt^ [10]b treatment group (0.18 ± 0.03) compared to the HUCMSCs^Emp^ treatment group (0.14 ± 0.08) (Figure 5b). Additionally, these effects were significantly inhibited by IWR-1 (Figure 5(a,b)). Next, we verified the effect of HUCMSCs^Wnt^ [10]b on the migration of HUVECs using the Transwell™ assay as described above and found that again, HUCMSCs^Wnt^ [10]b promoted the migration of HUVECs (Figure 5c). Furthermore, tube formation assay of HUVECs showed that HUCMSCs^Wnt^ [10]b-conditioned medium significantly improved the angiogenic ability of HUVECs (Figure 5d). The number of tubes (41.33 ± 1.85) and branch points (76.00 ± 2.44) in the HUCMSCs^Wnt^ [10]b group were both obviously higher than in the HUCMSCs^Emp^ group (29.33 ± 2.74 tubes and 21.00 ± 2.94 branch points) or any other groups (Figure 5(e,f)). These effects were abolished after the addition of *SU5416*, an angiogenesis inhibitor (Figure 5d, e and f). Combining those *in vivo* and *in vitro* data, we hypothesized that HUCMSCs^Wnt^ [10]b enhance vessel growth to promote the turnover of cartilage to bone, which is one important mechanism for accelerated fracture healing.

## 4 Discussion

Implantation of stem cells at the injury site is considered to be an effective method of promoting tissue regeneration [18,19]. They can not only directly differentiate and replace the damaged tissue [20], but also regulate endogenous tissue repair, such as by secreting bioactive factors to activate local stem cells, thereby indirectly promoting tissue regeneration [21,22]. Because Wnt is a factor that promotes osteogenesis and angiogenesis, we generated HUCMSCs^Wnt^ [10]b, hoping to achieve dual effects by both the secretion of Wnt and the provision of a source of stem cells to promote fracture healing.

In this study, we found that, compared with HUCMSCs^Emp^, HUCMSCs^Wnt^ [10]b significantly promoted fracture healing, especially by increasing the bone mass in the callus of the fractured site. The known effects of Wnt on osteogenesis and the marked difference between HUCMSCs^Emp^ and HUCMSCs^Wnt^ [10]b suggest that these therapeutic effects are caused by the Wnt10b protein secreted by HUCMSCs^Wnt^ [10]b acting on the endogenous stem cells of the femur, such as PSCs, rather than the osteogenic differentiation of HUCMSCs^Wnt^ [10]b themselves [23]. The healing of long bone fractures is a complex physiological process, which can be divided into three main stages: (1) formation of inflammatory hematoma after fracture injury; (2) activation and differentiation of bone stem/progenitor cells in the periosteum into chondrocytes to form cartilage callus, and with the growth of blood vessels, reconstructed the cartilage callus into a hard callus rich in osteoblast lineage cells; (3) activation of osteoclasts to reconstruct the callus and repair the fracture [3]. During fracture healing, the callus provides initial stability for the fractured site, and instability at the fracture site seems to be a major risk for development of nonunion [24]. Through the three-point bending tests, we found that HUCMSCs^Wnt^ [10]b significantly enhanced the mechanical properties of the fracture callus, possibly because they enhanced the migration and osteogenic differentiation of PSCs, and accelerated callus formation. Therefore, HUCMSCs^Wnt^ [10]b may be a precise target for the treatment of fracture nonunion.

Blood vessels allow the transport of cells, oxygen, nutrients, and waste, but also provide so-called vascular secretion signals that control organ growth and homeostasis [25]. During fracture healing, blood vessels play an important role in the formation of inflammatory hematoma and the transformation of cartilage callus into hard callus [26,27]. Consequently, angiogenesis disorder is one of the important causes of delayed healing or fracture nonunion [28]. Recently, it has been reported that activation of the Wnt/β-catenin pathway in vascular endothelial cells promotes the expression of vascular endothelial cell adhesion protein VE-cadherin, thereby promoting neovascularization [29]. In our study, we observed an increase of CD31+ blood vessels in the HUCMSCs^Wnt^ [10]b treatment group, and cytological experiments also showed that HUCMSCs^Wnt^ [10]b conditioned medium had a stronger effect in promoting vascular endothelial growth factor (VEGF)-mediated angiogenesis of HUVECs. These results indicated that HUCMSCs^Wnt^ [10]b promoted endothelial cell regeneration and angiogenesis, and thus promoted fracture healing. However, although HUCMSCs^Wnt^ [10]b can promote VEGF-mediated angiogenesis, exactly how they activate and regulate the VEGF pathway needs to be further elucidated.

## 5 Conclusion

In summary, our study demonstrates that HUCMSCs^Wnt^ [10]b promote fracture healing by accelerating hard callus formation, possibly by enhancing the osteogenic differentiation of PSCs and increasing blood vessel growth. These results suggest that implantation of HUCMSCs^Wnt^ [10]b may be a promising treatment for fractures.

## Supplementary Material

Supplemental Material

## References

[1] CHEN W, LV H, S LIU, et al. National incidence of traumatic fractures in China: a retrospective survey of 512 187 individuals [J]. The Lancet. Global Health. 2017;5(8):e807–e17. 28666814 10.1016/S2214-109X(17)30222-X

[2] Kostenuik P, M MIRZAF. Fracture healing physiology and the quest for therapies for delayed healing and nonunion [J]. J Orthop Res. 2017;35(2):213–223. 27743449 10.1002/jor.23460 PMC6120140

[3] A EINHORNT, C GERSTENFELDL. Fracture healing: mechanisms and interventions [J]. Nat Rev Rheumatol. 2015;11(1):45–54. 25266456 10.1038/nrrheum.2014.164 PMC4464690

[4] D HANKENSONK, Gagne K, Shaughnessy M. Extracellular signaling molecules to promote fracture healing and bone regeneration [J]. Adv Drug Deliv Rev. 2015;94(3):3–12. 26428617 10.1016/j.addr.2015.09.008

[5] Lanske B, Chandler H, PIERCE A, et al. Abaloparatide, a PTH receptor agonist with homology to PTHrP, enhances callus bridging and biomechanical properties in rats with femoral fracture [J]. J Orthop Res. 2019;37(4):812–820. 30790359 10.1002/jor.24254

[6] R HIXONK, A MCKENZIEJ, W SYKESDA, et al. Ablation of proliferating osteoblast lineage cells after fracture leads to atrophic nonunion in a mouse model [J]. J Bone Miner Res. 2021;36(11):2243–2257. 34405443 10.1002/jbmr.4424 PMC8719642

[7] WANG C, YING J, X NIE, et al. Targeting angiogenesis for fracture nonunion treatment in inflammatory disease [J]. Bone Res. 2021;9(1):29. 34099632 10.1038/s41413-021-00150-4 PMC8184936

[8] WANG T, ZHANG X, D BIKLED. Osteogenic differentiation of periosteal cells during fracture healing [J]. J Cell Physiol. 2017;232(5):913–921. 27731505 10.1002/jcp.25641 PMC5247290

[9] Debnath S, R YALLOWITZA, Mccormick J, et al. Discovery of a periosteal stem cell mediating intramembranous bone formation [J]. Nature. 2018;562(7725):133–139. 30250253 10.1038/s41586-018-0554-8 PMC6193396

[10] WANG L, J TOWERR, Chandra A, et al. Periosteal mesenchymal progenitor dysfunction and extraskeletally-derived fibrosis contribute to atrophic fracture nonunion [J]. J Bone Miner Res. 2019;34(3):520–532. 30602062 10.1002/jbmr.3626 PMC6508876

[11] Majidinia M, Sadeghpour A, Yousefi B. The roles of signaling pathways in bone repair and regeneration [J]. J Cell Physiol. 2018;233(4):2937–2948. 28590066 10.1002/jcp.26042

[12] HUANG P, R YAN, ZHANG X, et al. Activating Wnt/beta-catenin signaling pathway for disease therapy: challenges and opportunities [J]. Pharmacol Ther. 2019;196(79):79–90. 30468742 10.1016/j.pharmthera.2018.11.008

[13] Sarahrudi K, Thomas A, Albrecht C, et al. Strongly enhanced levels of sclerostin during human fracture healing [J]. J Orthop Res. 2012;30(10):1549–1555. 22508529 10.1002/jor.22129

[14] Y LIU, FANG J, ZHANG Q, et al. Wnt10b-overexpressing umbilical cord mesenchymal stem cells promote critical size rat calvarial defect healing by enhanced osteogenesis and VEGF-mediated angiogenesis [J]. J Orthop Translat. 2020;23:29–37. 10.1016/j.jot.2020.02.009 PMC7248289 32477867

[15] HUANG Q, Y ZOU, C ARNOM, et al. Hydrogel scaffolds for differentiation of adipose-derived stem cells [J]. Chem Soc Rev. 2017;46(20):6255–6275. 28816316 10.1039/c6cs00052e

[16] Leppanen O, Sievanen H, Jokihaara J, et al. Three-point bending of rat femur in the mediolateral direction: introduction and validation of a novel biomechanical testing protocol [J]. J Bone Miner Res. 2006;21(8):520–532. 16869721 10.1359/jbmr.060511

[17] CHEN W, Y SUN, GU X, et al. Conditioned medium of human bone marrow-derived stem cells promotes tendon-bone healing of the rotator cuff in a rat model [J]. Biomaterials. 2021;271(120714):120714. 33610048 10.1016/j.biomaterials.2021.120714

[18] P WONGS, E ROWLEYJ, N REDPATHA, et al. Pericytes, mesenchymal stem cells and their contributions to tissue repair [J]. Pharmacol Ther. 2015;151:107–120. 10.1016/j.pharmthera.2015.03.006 25827580

[19] ZHANG Y, Z HAO, WANG P, et al. Exosomes from human umbilical cord mesenchymal stem cells enhance fracture healing through HIF-1alpha-mediated promotion of angiogenesis in a rat model of stabilized fracture [J]. Cell Prolif. 2019;52(2):e12570. 30663158 10.1111/cpr.12570 PMC6496165

[20] G WALMSLEYG, C RANSOMR, R ZIELINSE, et al. Stem Cells in Bone Regeneration [J]. Stem Cell Rev Rep. 2016;12(5):524–529. 27250635 10.1007/s12015-016-9665-5 PMC5053855

[21] Bagno L, E HATZISTERGOSK, Balkan W, et al. Mesenchymal stem cell-based therapy for cardiovascular disease: progress and challenges [J]. Mol Ther. 2018;26(7):524–529. 29807782 10.1016/j.ymthe.2018.05.009 PMC6037203

[22] G PHINNEYD, F PITTENGERM. Concise review: MSC-derived exosomes for cell-free therapy [J]. Stem Cells. 2017;35(4):851–858. 28294454 10.1002/stem.2575

[23] Wilk K, A YEHS, J MORTENSENL, et al. Postnatal calvarial skeletal stem cells expressing PRX1 reside exclusively in the calvarial sutures and are required for bone regeneration [J]. Stem Cell Reports. 2017;8(4):933–946. 28366454 10.1016/j.stemcr.2017.03.002 PMC5390237

[24] Rupp M, Biehl C, Budak M, et al. Diaphyseal long bone nonunions - types, aetiology, economics, and treatment recommendations [J]. Int Orthop. 2018;42(2):247–258. 29273837 10.1007/s00264-017-3734-5

[25] P KUSUMBEA, K RAMASAMYS, H ADAMSR. Coupling of angiogenesis and osteogenesis by a specific vessel subtype in bone [J]. Nature. 2014;507(7492):323–328. 24646994 10.1038/nature13145 PMC4943525

[26] Peng Y, WU S, LI Y, et al. Type H blood vessels in bone modeling and remodeling [J]. Theranostics. 2020;10(1):426–436. 31903130 10.7150/thno.34126 PMC6929606

[27] H LIUJ, Yue T, W LUOZ, et al. Akkermansia muciniphila promotes type H vessel formation and bone fracture healing by reducing gut permeability and inflammation [J]. Dis Model Mech. 2020;13(11). DOI:10.1242/dmm.043620. PMC7725610 33033107

[28] C LIMJ, I KOK, Mattos M, et al. TNFalpha contributes to diabetes impaired angiogenesis in fracture healing [J]. Bone. 2017;99(26):26–38. 28285015 10.1016/j.bone.2017.02.014 PMC5563392

[29] Hubner K, Cabochette P, Dieguez-hurtado R, et al. Wnt/beta-catenin signaling regulates VE-cadherin-mediated anastomosis of brain capillaries by counteracting S1pr1 signaling [J]. Nat Commun. 2018;9(1):4860. 30451830 10.1038/s41467-018-07302-x PMC6242933

