# Supplementary figures and images for: Wnt10b-overexpressing umbilical cord mesenchymal stem cells promote fracture healing via accelerated cartilage callus to bone remodeling
Source: Bioengineered. 2022 Apr 18;13(4):10313–23. doi: 10.1080/21655979.2022.2062954 (PMC9161882; doi:10.1080/21655979.2022.2062954)

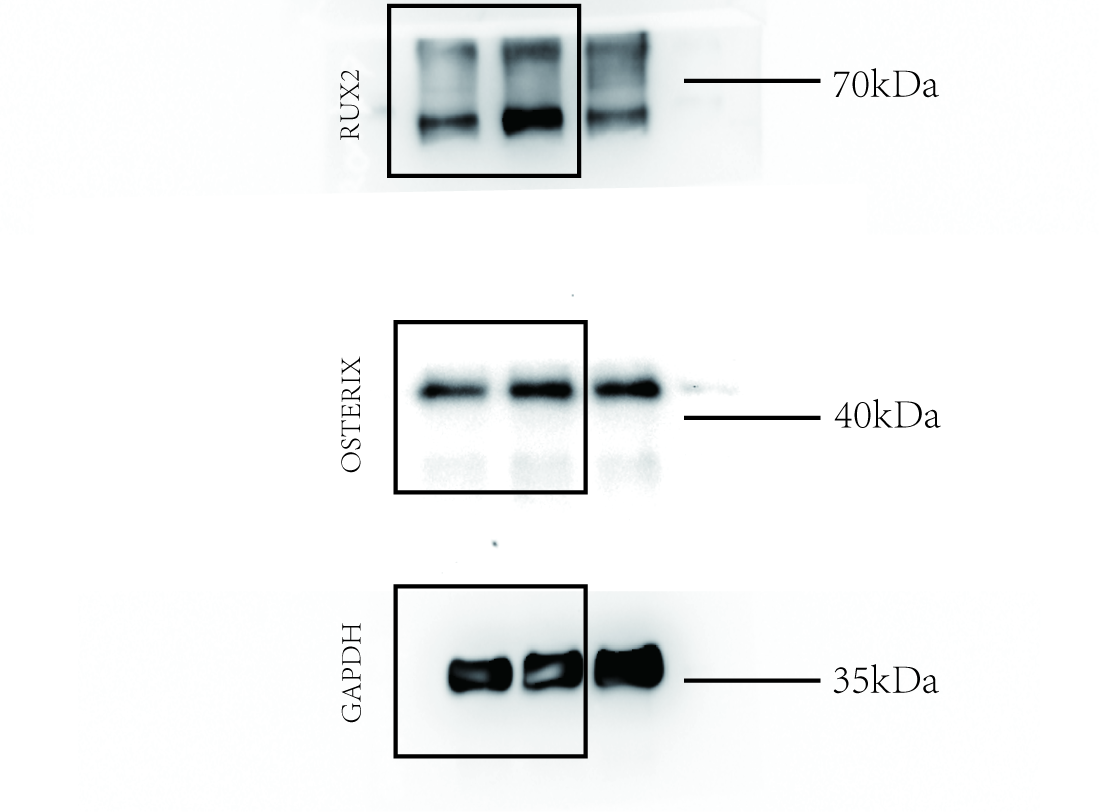

Supplement: Supplemental Material [file KBIE_A_2062954_SM9066.tif]
